# Thrombin Generation Induced by Complement Component C1q Interaction With the Receptor for the Globular Head of C1q on Adventitial Fibroblasts and Vascular Smooth Muscle Cells

**DOI:** 10.1002/iid3.70374

**Published:** 2026-03-07

**Authors:** Christopher Thor Freda, Wei Yin, Berhane Ghebrehiwet, David A. Rubenstein

**Affiliations:** ^1^ Department of Biomedical Engineering Stony Brook University Stony Brook New York USA; ^2^ Department of Medicine Stony Brook University Stony Brook New York USA

**Keywords:** complement, extrinsic coagulation, gC1qR, inflammation, tissue factor

## Abstract

**Introduction:**

Heightened inflammatory and thrombotic processes are common hallmarks of vascular diseases. The interaction between these two processes remains unclear and a better understanding of these links can allow for the design of more effective treatment options. Activation of complement component 1 (C1) leads to the initiation of the classical arm of the complement cascade, availability of plasma C1q, and the potential association of C1q and receptors for C1q. The association of C1q and gC1qR, the receptor for the globular head of C1q, is notable and has been associated with a wide range of disturbed physiological processes. We have recently shown that when this interaction occurs on vascular wall cells, including adventitial fibroblasts and vascular smooth muscle cells, there is a significant up‐regulation of tissue factor (TF) expression. However, whether or not this TF is biologically active and can facilitate extrinsic coagulation activation remains unknown. We hypothesized that TF expressed via gC1qR‐C1q association would support the progression of extrinsic coagulation.

**Methods:**

We quantified the association of Factor VII/VIIa (FVII/FVIIa) with adventitial fibroblast and vascular smooth muscle cell TF, using colorimetric assays. Further, we observed the formation of Factor Xa and Factor IIa (thrombin), as well as the concentration of intracellular Akt (protein kinase B) and phosphorylated Akt.

**Results/Conclusions:**

Our results indicate that TF expression in response to C1q exposure accelerates zymogen formation within the extrinsic coagulation cascade and alters Akt/p‐Akt expression. Overall, these findings highlight a significant connection between altered innate inflammation and heightened thrombin generation.

## Summary

Research reported in this publication was supported by the National Institute of Allergy and Infectious Disease of the National Institutes of Health under award number R21AI146535. The authors (BG) receive royalties from the sale of monoclonal antibodies against gC1qR clone 60.11. The authors (BG) hold a patent for the development of these antibodies for therapy against cancer and angioedema, respectively (US patent 8,883,153‐B2, “Methods for Prevention and Treatment of Angioedema”). The data that support the findings of this study are available from the corresponding author upon reasonable request.

## Graphical abstract

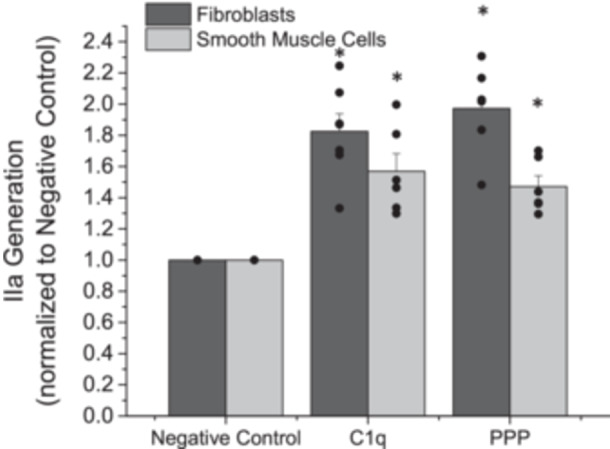

## 1 Introduction

Studies on the pathophysiology of vascular diseases have discovered numerous clinically observable complications, including disturbed inflammatory and thrombotic processes [1, 2]. One of the most common complications observed during vascular disease progression is plaque buildup within the vascular wall, or atherosclerosis, resulting in the narrowing of blood vessels and decrease in cardiovascular efficiency [3]. For many years, the known vascular complications associated with atherosclerosis were considered as the primary drivers of disease progression. However, more recent work has highlighted that alterations to both the inflammatory and thrombotic systems significantly contribute to atherosclerotic pathologies [4, 5]. Despite these findings, the interaction between the inflammatory and thrombotic systems remains unclear, even though complications of these systems are observed and well‐documented.

Physiologically, inflammatory processes occur as a defense mechanism in response to infection or cell injury, while hemostatic processes act to minimize blood loss, particularly during tissue damage. Both inflammatory and thrombotic processes can be triggered by exposure to cytokines, various chemical factors (e.g., the complement system), or tissue damage [6, 7]. However, an understanding of the role of innate inflammatory processes, including complement activities, on thrombosis, remains unclear. The complement system is a series of enzymatic reactions that result in the formation of the membrane attack complex and the lysis of pathogens [8]. Disturbed thrombosis can result in blood vessel obstruction and the unintended initiation of the intrinsic and extrinsic arms of the coagulation cascade [9]. Interactions between these two systems may lead to accelerated or new pathways for disease progression.

For many years, it has been known that complement activities can directly initiate intrinsic coagulation [10], however, the role of these process on extrinsic coagulation, the more physiologically‐relevant arm of coagulation, remanined unclear. We have recently reported a previously unknown pathway for the initiation of extrinsic coagulation via C1q association with gC1qR on the primary TF‐bearing cells, vascular smooth muscle cells (SMCs) and adventitial fibroblasts (AFs) [11]. In that report, we illustrate clear evidence that it is the interaction between C1q and gC1qR that alters downstream TF expression and by pharmacologically blocking this interaction TF expression stays at baseline conditions [11]. However, whether this new pathway for the initiation of extrinsic coagulation can lead to the common coagulation pathway and thrombin generation remained unclear. Thus, the aim of this work was to establish the significance of C1q induced TF expression.

C1q, complement component 1q, is one of the initiating components in the classical arm of the complement cascade. In addition to an association with antigen‐antibody complexes [12, 13], C1q has an affinity for gC1qR, a ubiquitous cell surface receptor that is found on cell types associated with cardiovascular disease development [14]. The binding of C1q to gC1qR has been associated with autoimmune diseases, intrinsic coagulation, endothelial cell activation, platelet activities, COVID‐19, and carcinogenesis [15, 16, 17, 18, 19, 20, 21, 22]. Complement component 1 (C1) is activated during early pathological cardiovascular events, leading to increased levels of circulating C1q [23], and deposition of C1q in the sub‐endothelial space. Sub‐endothelial C1q influences vascular smooth muscle cell functions related to proliferation and TF expression [11, 24]. Circulating FVII associates with TF to initiate extrinsic coagulation through Factor X activation, leading to thrombin generation. Given our findings on C1q's role in TF activation, we aim to determine if this TF is biologically active and capable of generating thrombin. Characterizing this pathway could open new avenues for vascular disease research and therapeutic interventions.

## 2 Materials and Methods

### 2.1 Cells

Human aortic adventitial fibroblasts (AFs) and human coronary artery smooth muscle cells (SMCs) were purchased from ScienCell Research Laboratories (Carlsbad, CA) and Cell Applications Inc. (San Diego, CA), respectively, and cultured as previously reported with the following modifications [11]. AFs were maintained in fibroblast medium‐2 supplemented with 5% fetal bovine serum, 1% antibiotics (penicillin/streptomycin), and 1% growth supplement (as suggested by ScienCell Research Laboratories), at 37°C and 5%CO_2_. SMCs were maintained in smooth muscle cell medium supplemented with 5% fetal calf serum, 1% antibiotics (penicillin/streptomycin), 0.1% insulin, and 0.2% growth supplements (as suggested by Cell Applications) at 37°C and 5%CO_2_. AFs and SMCs were cultured on tissue culture plastic flasks for propagation and well‐plates for experiments. Upon confluence, both cell types were passaged with trypsin digestion for approximately 2 min at room temperature (note that all reagents were purchased from Millipore Sigma, unless noted otherwise). All cells were maintained on standard tissue‐culture‐treated T‐75 flasks (for culture and maintenance) or 96‐well plates (experiments). During experiments, both cells were incubated with either 0.1 μg/mL purified human C1q (QuidelOrtho, San Diego, CA), lipopolysaccharides from *Escherichia coli* (LPS), or human platelet poor plasma (PPP, 1:10 in HEPES‐buffered Tyrode's solution, pH 7.4) for 60 min [11]. Platelet poor plasma is used as a positive control as it provides cells with access to all plasma proteins; providing cells with the ability to complete many inflammatory and thrombotic reactions, typically once activated. PPP was obtained from platelet rich plasma (Oklahoma Blood Institute, Oklahoma City, OK, USA) by centrifugation for 6 min at 3000 rpm (1100*g*). PPP samples can be stored at −80°C until use. LPS is used to agonize internal signaling within cells. All experiments also included an internal negative control consisting of cells exposed only to media for the entire duration. For statistical purposes, cell seeding density was maintained at ~40,000 cells/cm^2^ for all experiments. Note that all appropriate ethical guidelines were followed during this study.

### 2.2 Factor VIIa Association With Tissue Factor

To determine whether C1q mediated expressed TF is biologically active, we assessed the ability for TF to associate with exogenous FVII and the conversion from FVII to FVIIa. After cells were exposed to C1q, LPS, PPP, or no exogenous additives for 1 h, cells were washed twice with warmed PBS and then exposed to 10 nM FVII (in normal cell media) for 20 min at 37°C. After this exposure, cells were washed twice with warmed PBS and fixed with 0.5% glutaraldehyde (15 min, 37°C, pH 7.4). Cells were then washed twice (warmed PBS), and then 100 mM glycine –0.1% bovine serum albumin (30 min, room temperature, pH 7.4), was used to neutralize and block the samples. Samples were washed and then incubated with a primary antibody against FVIIa (Abcam, Waltham MA) for 1 h (room temperature), followed by incubation with an appropriate alkaline phosphatase conjugated secondary antibody. Color development was achieved via the addition of pNPP, and absorbance was observed spectrophotometrically at 405 nm (Molecular Devices, SpectraMax i3, this was used for all spectrophotometric measurements). All final measurements were conducted in a final volume of 60 μL, with at least two daily experimental replicates.

### 2.3 Factor Xa Generation

Association of FVII/FVIIa with C1q mediated expressed TF provides a potential pathway for extrinsic coagulation activation. To determine whether or not the TF—FVIIa complex is biologically active, we observed the ability for Factor Xa to be formed. As above, cell samples were exposed to experimental conditions (C1q, LPS, PPP, or no exogenous additives) for 1 h. Following this cells were washed twice in PBS and then exposed 10 nM FVII, 136 nM Factor X, and 5 mM CaCl_2_ for 20 min. Samples were incubated with 0.3 mM chromozym‐X (Roche, Basel, Switzerland) for 10 min and absorbance was observed spectrophotometrically at 405 nm. All final measurements were conducted in a final volume of 150 μL, with at least two daily experimental replicates.

### 2.4 Thrombin Generation

To quantify whether or not formed Factor Xa can mediate the conversion of prothrombin into thrombin, thrombin generation was assessed. Cells were incubated with experimental conditions for 1 h as above. Factor Xa was then generated by exposing cell samples to 10 nM FVII, 136 nM Factor X, and 5 mM CaCl_2_ for 20 min. Samples were removed and then incubated with 14.1μM prothrombin, 1μM Factor V, 5 mM CaCl_2_, and custom made phospholipid membranes (70% phosphatidylcholine and 30% phosphatidylserine by molar ratio, fabricated via sonication) for 5 min. After this incubation, samples were incubated with 0.3 mM chromozym‐Th (Roche) for 10 min and absorbance was observed spectrophotometrically at 405 nm. All final measurements were conducted in a final volume of 150 μL, with at least two daily experimental replicates.

### 2.5 Akt Quantification

To determine the potential internal mechanism responsible for C1q mediated TF expression, we evaluated the activity of the protein kinase B (Akt) pathway. After cells were treated with C1q, LPS, PPP, or no exogenous additives for 1 h, cells were washed twice with warmed PBS and fixed with 3.7% paraformaldehyde (15 min, 37°C, pH 7.4). Cells were then washed twice (warmed PBS), and neutralized and blocked with PBS + 1% BSA (30 min, room temperature, pH 7.4). This was followed by another PBS + 1% BSA (two times) and then cells were permeabilized with 0.2% Triton‐X (5 min). Cells were washed twice again with PBS + 1% BSA and then incubated with a primary antibody against either Akt (60203‐2‐Ig) or phosphorylated Akt on serine 473 (66444‐1‐Ig) (both from Proteintech, Rosemont, IL) for 90 min. After this incubation, cells were washed twice with PBS + 1% BSA and then incubated with a goat anti‐mouse Alexa Fluor 488 secondary antibody (A‐11001) (ThermoFisher Scientific, Waltham, MA). The cells were then washed twice with PBS and fluorescence was measured using a microplate reader to quantify the fluorescent signal from the secondary antibodies. The microplate reader was configured to excite the fluorescent tags at 485 nm and measure the intensity of the emitted fluorescence at 535 nm. All final measurements were conducted in a final volume of 60 μL, with at least two daily experimental replicates. Immediately after microplate measurements, fixed experimental samples were imaged on an inverted microscope (Nikon, TE‐2000U) for qualitative verification of quantitative immunofluorescence data. Camera exposure duration was held fixed during the verification.

### 2.6 Statistics

All experimental data from each independent experiment were normalized to the paired negative control (e.g., cells incubated without exogenous additives for the same duration). For most experiments, two to three dependent wells were assessed spectrophotometrically; these data were averaged prior to normalization. Normalized data from at least 5 independent experiments (sample size is reported in the figure legends) are shown and were used for statistical analyzes, which were conducted in SAS (v 9.4, SAS Institute, Cary, NC). Statistical significance was assessed with one‐way ANOVA procedures (the factor is in the incubation additive) with differences detected with the Tukey HSD post‐hoc test. For all experiments, α was set to 0.05. Note that all experiments were carried out following appropriate local regulations.

## 3 Results

### 3.1 Factors VII and VIIa

**Figure 1:**
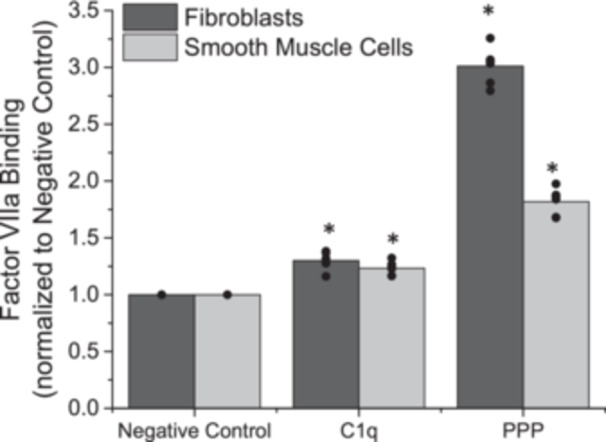
Normalized association of Factor VIIa on adventitial fibroblasts and coronary artery smooth muscle cells, following a 1 h exposure to C1q or platelet poor plasma (PPP). Negative control samples are paired experiments in the absence of exogenous additives. All data are reported as the mean + standard error of the mean from 6 independent experiments. * Significantly different than “Negative Control” (ANOVA, Tukey HSD method, *p* < 0.05). Independent data points are plotted as an overlay of the individual bars.

To determine whether or not the enhanced TF expressed due to the association of C1q with gC1qR on AFs and SMCs led to altered extrinsic coagulation, we exposed cells to FVII and quantified the amount of FVIIa associated with cells. The present FVIIa would likely be a component of the TF:FVIIa complex due to the favorable binding associations between these two molecules. The amount of FVIIa associated with AFs and SMCs increased significantly as compared with the negative control after exposure to C1q (Figure 1, *p* < 0.05). Platelet poor plasma (PPP), which serves as our positive control in these experiments, also significantly increased the quantity of FVIIa associated with AFs and SMCs.

### 3.2 Factor Xa

**Figure 2:**
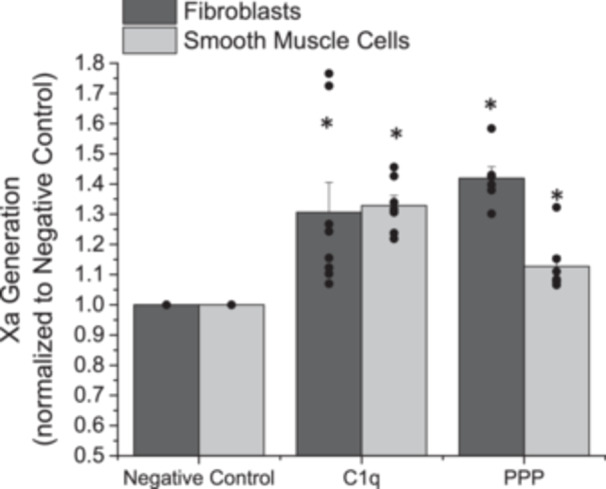
Normalized generation of Factor Xa after adventitial fibroblasts or coronary artery smooth muscle cells were exposed to C1q or platelet poor plasma (PPP) for 1 h. Negative control samples are paired experiments in the absence of exogenous additives. All data are reported as the mean + standard error of the mean from 6 to 8 independent experiments. * Significantly different than “Negative Control” (ANOVA, Tukey HSD method, *p* < 0.05). Independent data points are plotted as an overlay of the individual bars.

To determine if the formed TF:FVIIa complexes were biologically active and can lead to common coagulation initiation, we observed the formation of Factor Xa. As above, AFs and SMCs were independently exposed to C1q or PPP for 1 h. Cells were then provided access to FVII and Factor X, in the presence of calcium and the concentration of Factor Xa was assayed. The amount of Factor Xa generated was significantly increased after exposure to C1q or PPP as compared with the negative control (Figure 2, *p* < 0.05) for both AFs and SMCs. We observed an approximate 30% increase in Factor Xa generation under all observed conditions.

### 3.3 Thrombin

**Figure 3:**
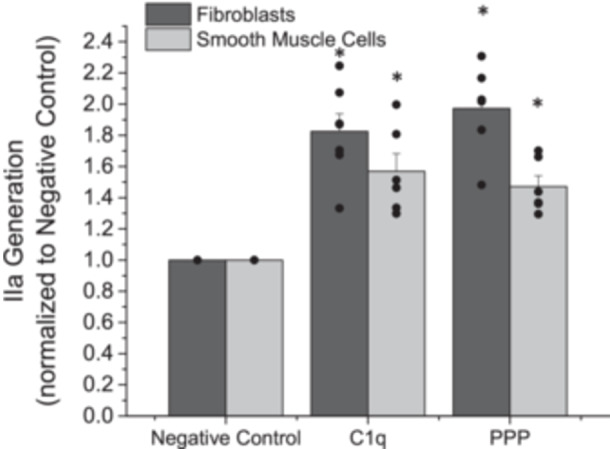
Normalized generation of thrombin (Factor IIa) after adventitial fibroblasts or coronary artery smooth muscle cells were exposed to C1q or platelet poor plasma (PPP) for 1 h. Negative control samples are paired experiments in the absence of exogenous additives. All data are reported as the mean + standard error of the mean from 6 to 7 independent experiments. * Significantly different than “Negative Control” (ANOVA, Tukey HSD method, *p* < 0.05). Independent data points are plotted as an overlay of the individual bars.

Similarly, we aimed to determine if formed Factor Xa was biologically active and can lead to enhanced thrombin generation. AFs and SMCs were independently exposed to C1q or PPP for 1 h and then incubated with appropriate coagulation complex components to monitor thrombin generation. The amount of thrombin generated was significantly increased after exposure to C1q or PPP as compared with the negative control (Figure 3, *p* < 0.05) for both AFs and SMCs. We observed an approximate 50%–90% increase in thrombin generation, depending on the incubation conditions.

### 3.4 Akt Signaling

**Figure 4:**
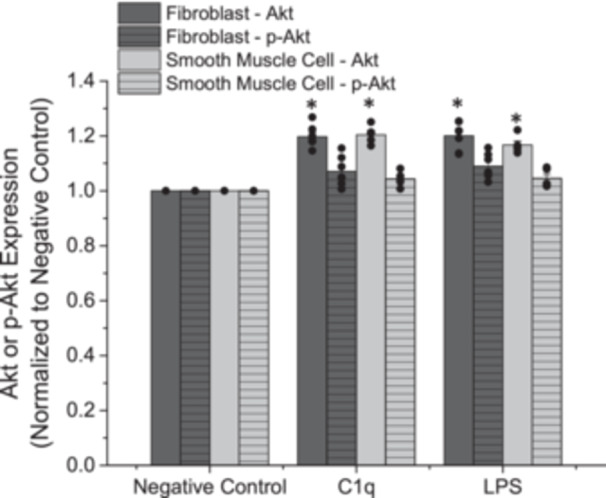
Normalized Akt/p‐Akt expression after adventitial fibroblasts or coronary artery smooth muscle cells were exposed to C1q or lipopolysaccharide (LPS) for 1 h. Negative control samples are paired experiments in the absence of exogenous additives. All data are reported as the mean + standard error of the mean from 5 to 7 independent experiments. * Significantly different than “Negative Control” (ANOVA, Tukey HSD method, *p* < 0.05). Independent data points are plotted as an overlay of the individual bars.

**Figure 5:**
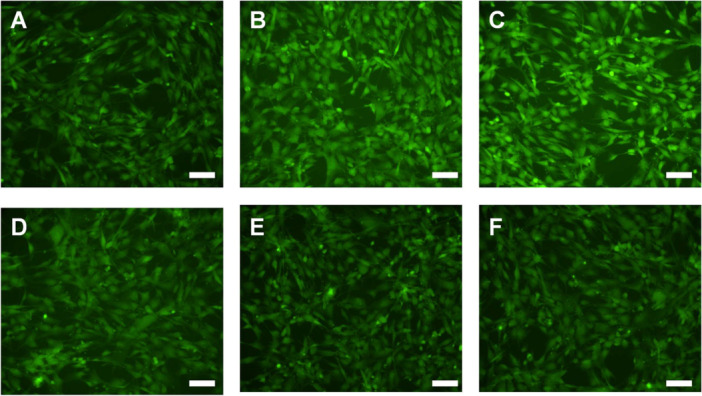
Representative images of Akt (A–C) or p‐Akt (D–F) expression in fibroblasts after exposure to control conditions (A and D), C1q (B and E), and LPS (C and F). These images provide confirmation of quantitative data shown in Figure 4. Scale bars are 100 μm.

Internal signaling pathways are important to determine, to help confirm underlying mechanisms responsible for the observed changes. The Akt pathway has been associated with enhanced gC1qR signaling and other innate inflammatory responses. As above, AFs and SMCs were independently exposed to C1q or LPS for 1 h and then Akt and p‐Akt concentrations were quantified. As compared with our negative control, the exposure to C1q significantly upregulated Akt concentrations in AFs and SMCs (Figure 4, *p* < 0.05). There was also an increase in p‐Akt under these conditions, however, the observed increase in p‐Akt did not achieve statistical significance. These increases mirrored the observed changes in response to our positive control, LPS. Qualitative validation of the data shown in Figure 4 is provided to show that C1q elicits a biological response consistent with our LPS positive controls (Figure 5). Further, the p‐Akt is present at a lower concentration than overall Akt.

## 4 Discussion

### 4.1 Factors VII and VIIa

We have previously reported an increase in TF expression after adventitial fibroblasts and vascular smooth muscle cells were exposed to C1q, which was mediated by gC1qR activity [11]. Here we aimed to determine if heightened expression would lead to an amplification of extrinsic and common coagulation reactions. We observed a significant increase in the activation of FVII to FVIIa, after exposure to C1q. Further, the formed FVIIa was associated with the expressed TF, which suggests that extrinsic coagulation could proceed towards the common coagulation pathway. This is an important new link that relates inflammatory activities to extrinsic coagulation activation. There have been extensive studies that report interactions between innate inflammation, including complement, and intrinsic coagulation [25, 26], however, connections between innate inflammation and extrinsic coagulation have not been investigated as extensively. Recent work has also exhibited that alterations to the activation of FVIIa can lead to enhancements of phosphatidylserine (PS) expression in endothelial cells and the release of PS enriched extracellular vesicles [27]. While downstream alterations to coagulation were not investigated in this model, it is well established that PS serves as a necessary primary docking site for many of the coagulation complexes and in the absence of PS containing vesicles/cells, the kinetic reaction rates of the coagulation enzymes are orders of magnitude lower than those in the presence of PS. Thus, observations on the activation of FVII and its downstream effects, as reported here, are critical to understanding downstream coagulopathies.

### 4.2 Factor Xa

Factor X activation is a critical step for common coagulation pathway activation and downstream activation of prothrombin. With increases in FVIIa formation and association with TF one would expect an increased Factor Xa generation, which has been observed under physiological and pathological conditions [28]. Here we show that with exposure to C1q, a potent innate inflammatory molecule, there was a significant increase in Factor Xa generation (Figure 2), as compared with cells not exposed to additional exogenous additives. To the best of our knowledge, no other group has looked at the role of complement activities on extrinsic coagulation activation leading to common coagulation. However, there has been work that illustrates that heightened inflammation can lead to altered common coagulation primarily through activation of the intrinsic coagulation cascade (summarized in [29]). While our work agrees with these previous findings, it also demonstrates that there is an alternative pathway that links inflammatory processes to common coagulation activation. Activation of both the intrinsic and extrinsic arms of the coagulation cascade can lead to significant pathological conditions that may overwhelm inhibitors processes or treatment options.

### 4.3 Thrombin

The formation of thrombin is significant during physiological and pathological bleeding. Thus, we aimed to determine if the observed changes in Factor Xa formation would alter the activation of prothrombin and form biologically active thrombin. After exposure to C1q, we observed a significant increase in thrombin generation (Figure 3), as compared with cells that were not exposed to additional exogenous additives. While it is well established that Factor Xa can mediate prothrombin activation, through both the intrinsic and extrinsic arms of coagulation, to the best of our knowledge this reaction has never been observed after C1q activation of extrinsic coagulation. Complement activities and altered thrombosis have been reported [10, 30, 31], and our work is in agreement with this and extends these findings.

### 4.4 Akt Signaling

To help characterize the observed changes in coagulation after exposure to C1q, we aimed to determine if the Akt signaling pathway was altered. Changes within the Akt signaling pathway have been associated with altered inflammatory potential and thus, this signaling marker makes a good first‐choice candidate to observe as a means to link inflammatory and thrombotic changes. We observed an increased expression of Akt and p‐Akt after exposure to C1q (Figure 4 and 5), as compared with cells not exposed to any exogenous additives. gC1qR has been shown to agonize the Akt signaling pathway during disease processes [32, 33]. Our findings illustrate that during disturbed inflammatory conditions, Akt can be up‐regulated and that this may relate to the enhanced TF expression and extrinsic coagulation observed.

## 5 Conclusions

The work presented here develops a further understanding of the interactions between the innate immune system and extrinsic coagulation. Links between these two systems are important to identify, so that better therapeutics and a more accurate understanding of physiological processes can be obtained. Our previous report illustrated that gC1qR‐C1q association was necessary for C1q‐mediated TF expression on adventitial fibroblasts and vascular smooth muscle cells. Here we show that this heightened TF expression can activate extrinsic coagulation leading to common pathway activation and thrombin formation.

## 6 Study Limitations

A limitation of the present study is that the experimental conditions employed were necessarily idealized and may not fully recapitulate the complexity of *in vivo* inflammatory and coagulation responses. In particular, the use of cell systems limits the ability to assess how additional cell types, mechanical forces, and circulating regulatory factors may modulate this pathway. Future studies employing more physiologically relevant models will be required to determine the extent to which gC1qR–C1q–mediated TF expression contributes to extrinsic coagulation under pathological conditions. Nevertheless, these findings underscore the importance of further defining the interface between inflammation and thrombosis to better inform therapeutic strategies.

## Author Contributions

Christopher Thor Freda was responsible for data curation, formal analysis, investigation, validation, visualization, and writing – original draft. Wei Yin was responsible for conceptualization, resources, and writing – review and editing. Berhane Ghebrehiwet was responsible for resources and writing – review and editing. David A. Rubenstein was responsible for conceptualization, formal analysis, funding acquisition, project administration, resources, supervision, and writing – review and editing.

## Conflicts of Interest

The authors (B.G.) receive royalties from the sale of monoclonal antibodies against gC1qR clone 60.11. The authors (B.G.) hold a patent for the development of these antibodies for therapy against cancer and angioedema, respectively (US patent 8,883,153‐B2, “Methods for Prevention and Treatment of Angioedema”).

## Data Availability

The data that support the findings of this study are available from the corresponding author upon reasonable request.
